# The 6th SASDiR Biennial Conference editorial

**DOI:** 10.4102/jamba.v17i2.2000

**Published:** 2025-10-20

**Authors:** Manta D. Nowbuth, Priscila Carvalho

**Affiliations:** 1 Department of Engineering, Faculty of Engineering, University of Mauritius, Reduit, Mauritius; 2 Energy Institute, UCL Bartlett School of Environment, Faculty of Energy and Resources, University College London, London, United Kingdom

In a world shaped by climate extremes, rapid urban expansion and fragile ecosystems, disaster risk reduction is no longer a choice. It is the proactive shield that transforms vulnerability into resilience, and chaos into preparedness. Investing into risk reduction implies building climate-resilient communities and societies, saving lives and ensuring a future where communities not only survive but thrive.

The 6th International Biennial Southern Africa Society for Disaster Reduction (SASDiR) Conference held from 21 August 2024 to 23 August 2024 at Ravenala Attitude Hotel, Balaclava, Mauritius, aimed at consolidating awareness and strengthening know-how transfer on disaster risk reduction in Africa. The event hosted four keynote addresses, one round table session, three side workshops, 36 technical presentations and 8 poster sessions. The event welcomed participants from GIZ Botswana, North-West University, South Africa, the Human Sciences Research Council, South Africa, the Sustainable Development Initiative, Malawi, the Northern Cape Department of Agriculture, Environmental Affairs, Waseda University, Japan, ESRI Southern Africa, University of London, UK, Université des Mascareignes, Mauritius, and the University of Mauritius.

The first keynote speaker, Mr. Nuvin Khedah, Director of the Land Drainage Authority, Mauritius, shared his experience on flood management in a small island. The second keynote speaker, Professor Shibayama Tomoya, Waseda University, Japan, talked about recent studies on Coastal Disaster Mitigation in some Asian countries. The third keynote speaker, Dr. Gatkuoth Kai, Regional Coordinator for Africa, UNDRR, took the audience through the topic of Disaster Risk Institutions in a fast-changing risk paradigm, and the fourth keynote speaker, Professor Shanghai Jiao from Tong University, China, shared her experience on high-efficient and net-zero emission marine power system based on Solid Oxide Fuel Cell/Gas Turbine (SOFC/GT) for shipping decarbonisation. All keynote speakers were self-funded and brought a wealth of expertise, commitment, and cross-regional perspectives that enriched the dialogue on disaster resilience and sustainable development.

Technical sessions addressed, among others, key topics such as applications of AI and machine learning (ML) in disaster risk reduction; role and potential of sustainable urban planning in disaster risk reduction; radar technology and its contribution to early warning; infrastructure resilience to impacts of climate change; information collection and decision-making skills among South African fire chiefs; building resilience to impacts of climate change through Indigenous Knowledge Systems and Education; importance of transdisciplinary approaches to reducing climate-related risks; lessons learnt from the African Union Bi-annual Reporting on the implementation of the SFDRR and POA targets and Insuring against infrastructure Quality and Reliability.

The success of the event was made possible through the invaluable contributions from several key institutions. These included Professor Dewald van Niekerk, Head of the African Centre for Disaster Studies, Potchefstroom Campus South Africa, and President of the SASDiR, the Disaster Risk Reduction and Resilience (DR3) project – a Belmont funded project, led by Professor Catalina Spataru, Professor of Global Energy and Resources, Director UCL Energy Institute and Head of Islands and Coastal Research Laboratory from the University College London – the Coalition for Disaster Resilient Infrastructure (CDRI) in India, North-West University in South Africa, and the Local Organising Committee from the University of Mauritius. Their collaborative efforts, technical expertise and commitment to advancing resilience and sustainability played a pivotal role in shaping meaningful dialogue and driving impactful outcomes.

